# Conceptualisation and development of the Conversational Health Literacy Assessment Tool (CHAT)

**DOI:** 10.1186/s12913-018-3037-6

**Published:** 2018-03-22

**Authors:** Jonathan O’Hara, Melanie Hawkins, Roy Batterham, Sarity Dodson, Richard H. Osborne, Alison Beauchamp

**Affiliations:** 1 Health Systems Improvement Unit, Centre for Population Health Research, School of Health and Social Development, Deakin University, Geelong, VIC 3220 Australia; 2 Faculty of Economics, Dhurakij Pundit University, Bangkok, Thailand; 3 The Fred Hollows Foundation, Melbourne, Australia; 4 Department of Rural Health, Monash University, Moe, Australia

**Keywords:** Conversational health literacy assessment tool, CHAT, Health literacy, HLQ, Patient-centred care, Clinical assessment

## Abstract

**Background:**

The aim of this study was to develop a tool to support health workers’ ability to identify patients’ multidimensional health literacy strengths and challenges. The tool was intended to be suitable for administration in healthcare settings where health workers must identify health literacy priorities as the basis for person-centred care.

**Methods:**

Development was based on a qualitative co-design process that used the Health Literacy Questionnaire (HLQ) as a framework to generate questions. Health workers were recruited to participate in an online consultation, a workshop, and two rounds of pilot testing.

**Results:**

Participating health workers identified and refined ten questions that target five areas of assessment: supportive professional relationships, supportive personal relationships, health information access and comprehension, current health behaviours, and health promotion barriers and support.

**Conclusions:**

Preliminary evidence suggests that application of the Conversational Health Literacy Assessment Tool (CHAT) can support health workers to better understand the health literacy challenges and supportive resources of their patients. As an integrated clinical process, the CHAT can supplement existing intake and assessment procedures across healthcare settings to give insight into patients’ circumstances so that decisions about care can be tailored to be more appropriate and effective.

## Background

The World Health Organization defines health literacy as “the cognitive and social skills which determine the motivation and ability of individuals to gain access to, understand and use information in ways which promote and maintain good health” [1]. Health literacy is a multidimensional concept that encompasses not only the ability to comprehend health information, but also includes the resources and skills to communicate and interact with healthcare providers and services to obtain, understand, make decisions about, and act upon health information [2–5]. It is increasingly recognised that difficulty in managing health and care relate not only to the health literacy of individuals, but is also influenced by their experiences with more or less user-friendly health information and health services [6]. Health literacy diversity is an issue that healthcare organisations and providers need to be aware of and respond to appropriately [3].

To date, health literacy measurement has mainly been operationalised as health-related literacy with a focus on language and numerical comprehension. This is often referred to as functional health literacy. Low health literacy has been associated with adverse health outcomes and behaviours, including decreased ability to self-care [7], differential use of healthcare [8], higher rates of avoidable hospital admissions [9], and less use of preventive health services [10]. A number of tools have been used by healthcare providers to screen for patients who may have difficulty comprehending health information: Short Test of Functional Health Literacy in Adults [11]; Newest Vital Sign [12]; and the Brief Health Literacy Screening Items [13]. These tools do not capture the multidimensional nature of health literacy.

Beyond functional health literacy abilities, there is a need to identify patients who are less able to communicate with healthcare providers about their health care needs. Patients who have difficulty interacting with healthcare providers are vulnerable to inequitable access to health services and poorer healthcare outcomes [14, 15]. This ability to communicate is particularly important for patients with complex or chronic conditions who benefit from effective, ongoing relationships with healthcare providers.

Several multidimensional measures of health literacy have been developed to assess a broader range of health literacy resources and abilities [16–18]. However, these were developed for research applications such as for surveys of groups or populations. The Health Literacy Questionnaire (HLQ) was developed in extensive consultation with community members, practitioners, and managers [19, 20]. It provides rich information that is potentially valuable for clinical practice and has a strong and reproducible theoretical structure that has been found to be robust in several studies in Australia [21] and in other cultures [22–24]. The HLQ captures health literacy across nine domains and has become an internationally-used measure for health service improvement and research. Table 1 shows the domains of the HLQ and text descriptors for each domain.

**Table 1:** Domains of The Health Literacy Questionnaire (HLQ) with text descriptors

HLQ scale	Information about health literacy strengths and challenges derived from items in each domain
1. Feeling understood and supported by healthcare providers	If a person has a relationship with one or more healthcare providers who they feel they can rely on and/or trust for advice about health.
2. Having sufficient information to manage my health	If a person feels they have the information they need to take care of their health, and if they feel they have the right information to manage their health.
3. Actively managing my health	If a person actively engages with managing their own health or takes a more passive approach to health management.
4. Social support for health	If a person has one or more friends or family members they feel they can rely on and/or trust for support with health management (e.g., day-to-day things such as taking medications, appointment attendance, and/or emotional support).
5. Appraisal of health information	If a person tends to accept most health information they hear or see, or if they tend to think critically about the information they receive and if it is right for them.
6. Ability to actively engage with healthcare providers	If a person finds it easy or difficult to communicate openly and effectively with health providers and to continue with discussions until they feel they have the information they need.
7. Navigating the healthcare system	If a person is aware of health services and health providers that are appropriate for their needs, and when to access them.
8. Ability to find good health information	If a person knows where to find health information when they need it, and if they feel confident and able to source this information.
9. Understanding health information well enough to know what to do	If a person finds it easy or difficult to understand and follow health information they are provided with.
10. Health beliefs and culture	If there are significant social and/or cultural beliefs that prevent or restrict your client from engaging with health services and care.

The individual domains of the HLQ (assessed with 4 to 5 questions) can be used to identify specific health literacy strengths and challenges. However, the HLQ was not designed as a clinical tool and the information that it provides may not be immediately actionable. Nevertheless, the nine domains provide a strong foundation on which to base the development of a new health literacy tool.

At present, there is no multidimensional health literacy tool that allows health workers to quickly gain insight into their patients’ health literacy in a clinical setting. A tool that supports conversations about health literacy between a health worker and a patient could improve two-way communication and facilitate a more responsive and equitable approach to assessment. Such a tool could complement existing intake and assessment processes by providing early insight into a patient’s strengths and challenges across critical areas of health literacy, enabling better-informed and collaborative choices for care, and potentially identifying early interventions. Opportunities for more patient-centred approaches to care arise when providers understand the circumstances in which their patients take action on health information [4].

The aim of this study was to develop a health literacy tool to easily and systematically identify relevant information about patients’ individual health literacy strengths and challenges. Based on the domains of the HLQ, the tool was designed to support health workers to use a series of open-ended questions to have a structured conversation with patients. Qualitative information from these conversations was intended to be used by health workers to make targeted decisions in order to tailor care and determine opportunity for intervention. This tool was designed for use by health workers during routine interactions between patients seeking and receiving care across varying services (e.g., service intake or initial assessment) and settings (e.g., primary care, community or specialist treatment).

## Method

### Procedures

Development of the health literacy tool was based on a qualitative co-design process. A wide range of health workers (e.g., clinicians, paramedical staff, community health workers, and case managers) were involved to help ensure that the tool was appropriate for its intended purpose. The nine domains of the HLQ [20] were used as the a priori model to guide the generation of candidate questions and serves as a way to avoid missing potentially useful health literacy constructs. An additional domain representing health beliefs and culture was added to the HLQ domains. During the initial HLQ development this domain was identified as highly relevant to the construct of health literacy [20].

#### Stage 1: E-consultation

Health workers were invited to participate in an online consultation process. Participants were recruited across Home and Community Care providers in Victoria, and nationally via professional networks and social media. For each HLQ domain, participants were prompted with a brief descriptor and asked what questions they might use with patients to detect strengths and challenges and determine further details about this aspect of health literacy (see Table 1). Additional descriptors were included to represent health beliefs and culture.

The candidate questions from the e-consultation process were reviewed by the research team in two rounds (JO, MH, AB). In the first round, the candidate questions were evaluated to ensure that they clearly represented the health literacy constructs underlying each HLQ domain, and determined if questions were suitable for detecting health literacy strengths and challenges, or for determining further details about these strengths and challenges. In the second round, questions were excluded if they were context-specific, disease-specific, or were duplicates. After each round, the research team established consensus on which candidate questions to retain.

#### Stage 2: Workshop

Health workers from a range of settings and backgrounds were invited to participate in a two-hour workshop to examine and discuss the responses collected from the e-consultation. Participants were recruited via existing organisational collaborations, including e-consultation participants who expressed an interest in attending the workshop. During the workshop, three questions were discussed using a nominal group method: *Which domains of the HLQ are most clinically relevant to your patients?*; *What difficulties do you anticipate asking questions like these with your patients?*; and *What recommendations would you have for a new health literacy assessment tool?* Participants were asked to provide their views about the merits of each the questions derived from stage 1.

#### Stage 3: Initial pilot testing

Participating organisations were asked to recruit health workers through an internal expression of interest process. Participants were asked to administer the draft tool to at least two patients over a two-week period. Following this, the researchers conducted interviews with each provider and asked: *Describe what you think you learned from asking these questions;* and *In what way could the questions, or the process for asking them, be improved?* Based on findings from the interview data, the questions and supporting resources of the draft health literacy tool were refined for further testing.

#### Stage 4: Further pilot testing

Participating organisations again recruited health workers through an internal expression of interest process. Participating health workers were provided with written documentation, a video-based introduction, and paper-based forms to record patient responses. Participants were asked to recruit up to six patients over a period of 2 months, including both new and known patients. Inclusion criteria required that patients were over the age of 18, without cognitive impairment, and able to speak English. Semi-structured interviews conducted after further pilot testing asked health workers about their perceptions of the tool’s utility, how the information gathered would be used, if there were any practical issues that would limit its broader adoption, and if participants would recommend it to other health workers.

## Results

### Stage 1: E-consultation

Fifty-five health workers acting from community health services (73%), city councils (13%), and other health service providers (14%) participated in the e-consultation. Participants were located in Victoria (91%), or other Australian states and reported between 1 and 38 years of experience (median = 13). Collectively, participants submitted 364 questions they would use to enquire about health literacy with their patients. Of these questions, 198 were for the purpose of detecting health literacy strengths and challenges and 166 were for determining further details about these strengths and challenges. From these submissions, 100 unique questions were identified across the nine HLQ domains and the construct representing health beliefs and culture.

### Stage 2: Workshop

There were eight workshop participants. Roles represented included health promotion (*n* = 2), counselling (*n* = 2), health assessment (*n* = 2), occupational therapy (*n* = 1), and nursing (*n* = 1). All workshop participants expressed agreement that six HLQ domains would provide the most clinically-relevant information. Domain 2. Having sufficient information to manage my health was considered to be too general to be relevant in the context of a time-limited clinical encounter, and given the patient is in a consultation with a health worker who will provide more information if needed. Domain 5. Appraisal of health information was seen as a higher order skill that is desirable but not essential for supporting healthcare outcomes. Domain 7. Navigating the healthcare system was considered to be less relevant because patients would already be connected with a healthcare provider at the time of administration. As an exception, participants’ appraisal of the health beliefs and values construct was mixed, however it was retained for initial pilot testing.

Workshop participants provided feedback on which individual questions from Stage 1 would be most useful for assessing each health literacy domain. Some participants reported concerns about patients responding positively to questions in order to avoid detection of potential health literacy issues. Multiple participants noted cases of patients providing inconsistent answers at interview for this reason. To minimise this possibility, it was suggested that open-ended questions be used to explore patients’ routine health behaviours in order to provide information on their health circumstances and reveal their understanding of their own health needs. Additionally, participants suggested specifically asking about patients’ experiences of barriers and supports when accessing, understanding, and using health information and services.

Based on these findings, a draft tool with supporting documentation was prepared for initial pilot testing. This tool was named the Conversational Health Literacy Assessment Tool (CHAT) and featured 13 questions across five areas of assessment. Using three questions, section 1 targeted supportive professional relationships (addressing HLQ domains 1. Healthcare provider support and 6. Ability to actively engage with healthcare providers). Using two questions, section 2 reflected supportive personal relationships (addressing HLQ domain 4. Social support for health). With two questions, section 3 represented access to and understanding of health information (addressing HLQ domains 8. Ability to find good health information and 9. Understand health information well enough to know what to do). Using two questions, section 4 evaluated limitations due to health beliefs and values. Lastly, using four questions, section 5 enquired about the health behaviours of patients on a regular daily, weekly, and yearly basis, followed by a question about general supports and barriers: “*What is going well for you and what is harder to keep up on a regular basis?”*

### Stage 3: Initial pilot testing (13-item draft tool)

Nine health workers participated in the initial CHAT pilot testing and interviews. All nine were from community health services. Their roles included community health nursing (*n* = 3), occupational therapy (*n* = 2), physiotherapy (*n* = 2), clinical psychology (*n* = 1), and community dietetics (n = 1).

Several changes to the CHAT questions were made based on feedback from these interviews. Participants helped clarify the intention of each question by improving the expression. Both of the questions targeting health beliefs and values were removed because participants unanimously agreed that they were not immediately useful in the context of CHAT administration. For section 5, two participants noted that the yearly health behaviour question provided no value beyond the daily and weekly behaviour questions, and this was subsequently removed. One participant reported that some patients required prompting about health behaviours so supplementary prompts were added to guide conversation for these questions. Finally, the question regarding barriers and supports was divided into two discrete questions to streamline administration. Participants noted the value of closing the assessment positively with a question that highlighted patient’s perceived supports. The complete list of CHAT questions based on three stages of participant consultation is presented in Table 2.

**Table 2:** Conversational Health Literacy Assessment Tool (CHAT) Questions

Supportive professional relationships	1. Who do you usually see to help you look after your health?
	2. How difficult is it for you to speak with *[that provider]* about your health?
Supportive personal relationships	3. Aside from healthcare providers, who else do you talk with about your health?
	4. How comfortable are you to ask *[that person]* for help if you need it?
Health information access and comprehension	5. Where else do you get health information that you trust?
	6. How difficult is it for you to understand information about your health?
Current health behaviours	7. What do you do to look after your health on a daily basis? (Prompt for diet, sleeping habits, medication, and treatment plan)
	8. What do you do to look after your health on a weekly basis? (Prompt for exercise, physical activities, social activities, and visits to healthcare professionals)
Health promotion barriers and support	9. Thinking about the things you do to look after your health, what is difficult for you to keep doing on a regular basis?
	10. Thinking about the things you do to look after your health, what is going well for you?

### Stage 4: Further pilot testing (10-item tool)

Thirteen health workers represented a range of services, including community health nursing (*n* = 3), physiotherapy (*n* = 3), occupational therapy (*n* = 3), case management (diversional therapy, social work; *n* = 2), clinical psychology (*n* = 1), and podiatry (*n* = 1). Participants reported working in their respective fields from 1 to 33 years (median = 17). In total, participants conducted 46 CHAT interview sessions with patients (median = 3, range = 1–5). Participants used the CHAT exclusively with new patients (38%), a mix of existing and new patients (38%), and existing patients only (24%).

Interviews explored how the CHAT questions could be used to obtain detailed information about patients’ health literacy strengths and challenges. Across CHAT questions, participants reported conversations that revealed issues such as patients who lack sources of social support and experience isolation despite assistance from social workers; where patients find health information and how much they trust those sources; ‘disconnects’ between patient health knowledge and behaviours; and factors that make it harder for patients to self-manage their health on an ongoing basis.

Question 6, which is about understanding health information, was reported as the most valuable CHAT question overall. Participants reported extended conversations with their patients about this question, particularly with those who were self-managing their condition: *“It was really valuable to understand that clinicians need to spend enough time to help patients understand information that they are making decisions with… especially those who were trying to help themselves. Looking at how clinicians provide information to them and if there are other ways that clinicians could provide that information.”*

A trend emerged whereby the perceived utility of the remaining CHAT questions differed according to health service context. Health workers who were engaged in development of care plans in rehabilitation settings or with patients who have chronic health conditions, had more interest in the questions that revealed a patient’s broader health context and circumstances. Personal relationships (questions 3 and 4), and barriers and supports (questions 9 and 10) were all reported as providing valuable information for health outcomes that require long-term planning, strategy, and goal setting.

The benefit of understanding how patients find health information was unclear in contexts where obtaining further information was unnecessary. For example, within patient sessions that concluded with a request for a patient to simply follow guidance for care. Similarly, the benefit of understanding the presence and utility of professional relationships (questions 1 and 2) was less evident for patients who were already engaged with appropriate healthcare providers and felt no need for further assistance.

Mixed responses were reported for the questions about current health behaviours (questions 7 and 8). These questions provide additional information about the patient’s health context and circumstances but were reported as being less immediately beneficial for health workers than the questions that target barriers and supports. While one participant reported that these questions were an improvement on current assessment questions, two participants noted that these questions did not provide much new information when working with patients with whom they had an existing relationship.

When discussing the practicality of the CHAT, participants reported that adopting the tool into their regular procedures was straightforward: *“When I saw the questions, I felt quite comfortable that they would be easy to ask and administer without being confrontational, without making the patient feel like you are assessing their reading ability, numerical, and some of those other things”.* Accommodating the length of administration time with other competing demands was the key noted limitation to adoption.

Overall, participating health workers expressed a clear need for such a tool. One provider noted that the CHAT was needed *“...because for a lot of clinicians, they don’t know how to start the conversation. So I think it has got some useful questions for people who haven't developed that comfort to address all of those questions*”. When asked if they would use patients’ responses to the CHAT in their practice, the majority of participants (69%) said they would refer to the information during a review of a patient’s care plan or at discharge, depending on the healthcare service provided. Almost all participants (85%) reported that they would recommend the CHAT to other health workers, particularly those with less experience.

## Discussion

Preliminary evidence has demonstrated that the CHAT functions well as a brief assessment of health literacy within healthcare environments. Unlike numeric questionnaires, which can be difficult to interpret for immediate decision making, the ten CHAT questions can support health workers to engage in conversations with patients about specific health literacy strengths and challenges. The information that health workers learn about patients in such encounters can help them to tailor care to leverage the patient’s health literacy strengths or to mitigate the patient’s health literacy challenges. Nevertheless, despite the promising evidence from this initial study, further evaluation of the utility of the CHAT is warranted.

Overall, the CHAT questions address six of the nine HLQ domains, including domains 1. Feeling understood and supported by healthcare providers (CHAT question 1), 3. Actively managing my health (questions 7 and 8), 4. Social support for health (questions 3 and 4), 6. Ability to actively engage with healthcare providers (question 2), 8. Ability to find good health information (question 5), and 9. Understanding health information well enough to know what to do (question 6). Three HLQ domains are not addressed by the CHAT (Domains 2. Having sufficient information to manage my health, 5. Appraisal of health information, and 7. Navigating the healthcare system). Health workers described these domains as a lower priority in the context in which they engage patients.

Four CHAT questions identify the presence and quality of supportive resources that could be utilised in interventions to overcome identified health literacy challenges. These determine if patients are actively engaged with health service providers and with others socially (question 1 and 3), and whether there is opportunity to utilise these supportive relationships for practical support (question 2 and 4). Two questions assess specific health literacy needs regarding the access and understanding of health information (questions 5 and 6).

CHAT questions related to current health behaviours (question 7 and 8) provide broader context to a patient’s circumstances. These questions function similarly to the teach-back method of education, allowing patients to describe their regular health routines in their own words [25]. Lastly, two questions directly enquire about patient’s health promotion barriers and supports (question 9 and 10). Responses provided to these questions can help health workers to capture any further issues to be accommodated in order to achieve successful health outcomes for a patient.

### Practice implications

The CHAT offers potential benefits to patients, health workers, and healthcare organisations. Firstly, due to its conversational approach, CHAT offers an equitable means of health literacy assessment that can accommodate patients with diverse needs, literacy abilities, and levels of education. Secondly, the conversational approach of the CHAT promotes open communication and the development of stronger rapport, supporting positive healthcare outcomes [15]. Use of the CHAT may also support junior health workers to better engage with their patients. Thirdly, the CHAT has the potential to improve health workers’ insights into their patients’ diverse health literacy challenges and subsequent healthcare consequences, thus supporting healthcare organisations to be more responsive to the health literacy needs of their patients [6]. Finally, the CHAT can easily be integrated into existing assessment procedures, either in full or in part. Health workers may selectively apply the CHAT questions depending on the information that is likely to be most useful in their specific healthcare setting and context, given the limitations of administration time. Furthermore, it is envisaged that the patient engagement process embodied in the CHAT could be incorporated into habitual good practice, rather than being a stand-alone tool.

This study had two key strengths. Development of the CHAT was based on a previously established and robust conceptual framework, and use of a co-design approach helped to ensure that the tool met the needs of health workers. The primary limitation of this study is that the utility of the CHAT is yet to be fully assessed. More extensive assessment would explore if, and how, health workers are able to use information generated by the tool to tailor their decision making to improve care and treatment for patients.

Future research can explore the relevance of the five CHAT assessment areas across specific healthcare service contexts, such as acute care settings. CHAT questions regarding current health behaviours were most useful with new patients and within healthcare contexts which require greater understanding of a patient’s broader health behaviours, e.g. chronic conditions. Likewise, the question related to accessing trusted health information was not as useful for patients who did not require additional information to support their treatment or care.

## Conclusion

Across ten patient-centred questions, the CHAT generates information about the context and circumstances in which people manage their health. This includes details about resources, abilities, supports, and barriers that determine an individual’s ability to understand, access and use health information and services: that is, their health literacy. Health workers can use these insights to develop practical ideas about ways to assist individuals with different health literacy needs. The CHAT fills a gap in existing health literacy tools and it is hoped that it will assist health workers to achieve better and more equitable health outcomes for their patients.

## Abbreviations

CHAT: Conversational Health Literacy Assessment Tool
HLQ: Health Literacy Questionnaire
